# The complete chloroplast genome sequence of *Populus deltoides* ‘Siyang-2’

**DOI:** 10.1080/23802359.2019.1700840

**Published:** 2019-12-13

**Authors:** Tao Su, Mei Han, Jie Min, Dan Cao, Huixin Pan, Yuxin Liu

**Affiliations:** a Co-Innovation Center for Sustainable Forestry in Southern China, College of Biology and the Environment, Nanjing Forestry University, Nanjing, China;; b Key Laboratory of State Forestry Administration on Subtropical Forest Biodiversity Conservation, Nanjing Forestry University, Nanjing, China;; c College of Forestry, Nanjing Forestry University, Nanjing, China

**Keywords:** Populus deltoides ‘Siyang-2’, complete chloroplast genome, plastid, improved varieties

## Abstract

*Populus deltoides* ‘Siyang-2’ is an improved variety of forest trees that have been identified recently. It shows superior growth performance compared to other local cultivars in the region of Yangtze-Huaihe in China. In this study, the whole chloroplast (cp) genome sequence of ‘Siyang-2’ was assembled and characterized by high-throughput sequencing data. The complete cp genome was 156,957 bp in length, containing a large single-copy region (LSC) of 85,096 bp, and a small single-copy region (SSC) of 16,563 bp, which were separated by a pair of 27,649 bp inverted repeat regions (IRs). The cp genome contained 131 genes, including 86 protein-coding genes, 37 tRNA genes, and 8 ribosomal RNA genes. Most of the gene species occur as a single copy, while 20 gene species occur in double copies. The overall GC content of ‘Siyang-2’ cp genome is 34.6%, while the corresponding values of the LSC, SSC, and IR regions are 34.5%, 30.6%, 41.9%, respectively. The complete cp genome provides valuable phylogenetic and cp genetic engineering studies of this important improved poplar species *P. deltoides* ‘Siyang-2’.

*Populus deltoides* ‘Siyang-2’ is a selection breeding variety of *Populus*, developed by Dr. huixin Pan from the Poplar Research and Development Center in Nanlin (Nanjing Forestry University). This variety was authorized to be an improved variety of forest trees (Identifier: Su S-SC-PD-002-2018, http://lyj.jiangsu.gov.cn/art/2018/12/12/art_48797_7951901.html) by national forestry and grassland administration in China (Wang et al. 2019). ‘Siyang-2’ showed superior growth performance in comparison with ‘Nanlin 895’, an elite hybrid poplar clone in south China being widely planted for agricultural industry and scientific research (Fang and Yang 2003; Fang et al. 2006; Chai et al. 2014; Wang et al. 2016; Chao et al. 2019; Zhu et al. 2018). Although it showed great potential in the economy and ecosystem construction in south China, as a new variety, ‘Siyang-2’ has not been intensively studied yet. The genetic information and the phylogenetic relationship of ‘Siyang-2’ remained elusive. In this study, the complete chloroplast (cp) genome sequence of ‘Siyang-2’ was initially assembled and characterized.

The fresh leaves of *P. deltoides* ‘Siyang-2’ were sampled from the clonal trials in Baguazhou Farm, Nanjing, China (32°09′N, 118°49′E), and used for DNA extraction by DNeasy Plant Mini Kit (Qiagen, Valencia, CA, USA). The whole genome sequencing was implemented by Hefei Biodata Biotechnologies Inc. (Hefei, China) on the BGISEQ-500 platform. In total, about 34 MB high-quality clean reads were obtained. The filtered sequences were assembled using the program SPAdes assembler 3.10.0 (Bankevich et al. 2012). The resulting contigs were linked based on overlapping regions using *P. trichocarpa* (EF489041) (Tuskan et al. 2006) as reference. Annotation was performed using the DOGMA (Wyman et al. 2004) and BLAST searches.

The complete chloroplast/plastid genome sequence together with gene annotations was submitted to GenBank under the accession numbers of MN417118 for *P. deltoides* ‘Siyang-2’. The cp genome was determined to comprise double-stranded, circular DNA of 156,957 bp, including two inverted repeat (IR) regions of 27,649 bp each, separated by a large single-copy (LSC) and a small single-copy (SSC) region of 85,096 and 16,563 bp, respectively. The genome contained 131 genes, including 86 protein-coding genes, 37 tRNA genes, and 8 rRNA genes. One rps12 gene was divided into two independent transcription units. Most of the genes occurred as a single-copy, while eight protein-coding genes, four rRNA genes, and eight tRNA genes had two copies, respectively. In addition, three protein-coding genes had three exons each. Eight protein-coding genes and six tRNA genes contained two exons. The overall GC content is 34.6%, and the corresponding values in LSC, SSC, and IR regions are 34.5, 30.6, 41.9%, respectively.

The phylogenetic relationships of ‘Siyang-2’ with other 61 species in the Salicaceae family were analyzed by using MAFFT v7.307 (Katoh and Standley 2013). The ML phylogenetic tree (constructed by FastTree version 2.1.10 (Price et al. 2010)) showed that ‘Siyang-2’ was closely related to *P. deltoids* and *P. x Canadensis* (Figure 1). The cp genome provides valuable information for further population, phylogenetic, physiological, cp genetic engineering studies of this important improved poplar species *P. deltoides* ‘Siyang-2’.

**Figure 1.**
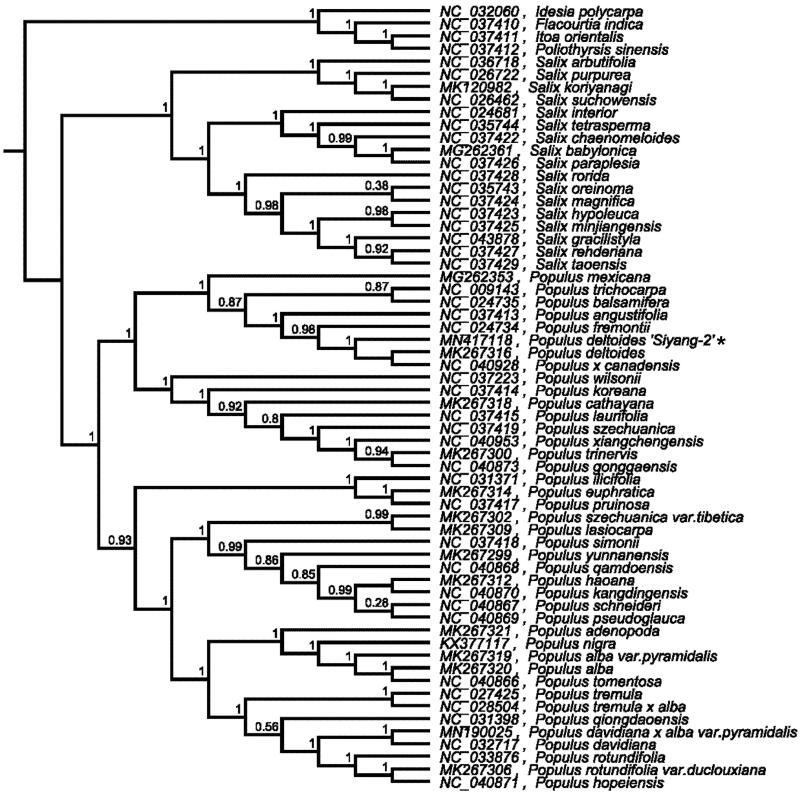
Phylogenetic tree inferred by maximum likelihood (ML) method based on the complete chloroplast genome of *P. deltoides* ‘Siyang-2’ and other 61 species in the *salicaceae* family, bootstrap values are shown on the branch.
